# Study of the Removal Efficiency of Chromium Ions Using a Membrane by Electro-Kinetic Technique from Sludge

**DOI:** 10.3390/membranes13090806

**Published:** 2023-09-21

**Authors:** Nabaa S. Hadi, Huda H. Awadh

**Affiliations:** Department of Environmental Engineering, College of Engineering, University of Babylon, Babil 51001, Iraq; eng.huda.ali@uobabylon.edu.iq

**Keywords:** chromium ions, contaminated sludge, electro-kinetic remediation, fixed anode, membrane

## Abstract

Recently, electro-kinetic (EK) remediation has become more popular as a novel method for removing chromium contamination from soil. This approach, however, is ineffective since it uses both cationic and anionic forms of chromium. In this study, a membrane-based technique was employed to increase the efficiency of the electro-kinetic removal of chromium. Chromium removal from polluted sludge was studied using four bench-scale experiments. Two of these experiments employed distilled water (EK−1 and EK−2 and membrane), whereas the other used acetic acid as the catholyte (EK−3 and EK−4 and membrane). The pH, total chromium, and fractionation of chromium in the sludge were measured after remediation. In the EK−1, EK−2 and membrane, and EK−3 and EK−4 and membrane trials, the average removal efficiencies of total chromium were 47.6%, 58.6%, and 74.4%, 79.6%, respectively. In contrast to the electro-kinetic remediation strategy, which left approximately 80% of the sludge neutral or alkaline after treatment, the membrane created acidic soil conditions throughout the sludge. For example, the high field intensity used in the membrane tests may have helped to facilitate chromium desorption, dissolution, and separation from the sludge and enhanced chromium mobility. The findings show that the membrane can improve the effectiveness of chromium removal from sludge when utilized in the EK remediation process.

## 1. Introduction

Strategies aimed at remediation, which involve the use of electrical DC fields to introduce transport mechanisms into the soil, are commonly known as electro-kinetic methods. It is possible to use electro-kinetics to transport ions, water, and charged particles via electromigration (EM), electro-osmosis (EO), and electrophoresis (EP) [1,2]. When conventional remediation techniques appear to have failed with fine-grained soils, electro-kinetic offers a significant advantage [3]. Single or combined processes require considerable electric fields (1–1.5 V/cm) to facilitate the migration of metal ions, resulting in the electrolytic breakdown of water and electrode wear. Thus, we can effectively lower energy consumption and costs by reducing the voltage used in electrochemical soil remediation methods [4].

This technique can be used on-site (in situ) or to excavate contaminated soil and transfer it to the laboratory (ex situ) [5,6]. The major feature of the electro-kinetic methods is the removal of in situ soil contaminants, which may be considered a preferred treatment choice compared to other conventional ex situ treatment methods, such as solidification/stabilization and soil washing [7,8,9,10]. Heavy metals accumulate in the pH leaping zone during the electro-kinetic remediation process. The ability of electro-kinetic remediation to remove heavy metals from soil is hampered by the accumulation effect. It has been observed that various strategies for improving the mobility of heavy metals may help reduce this limiting issue. Recently, electro-kinetic remediation of anodes has become a popular method in addition to chelating complexing chemicals. Soil acidification has been utilized to increase the buildup of heavy metals, according to previous reports [11,12].

The electro-kinetic experiment compared the influence of fixed electrodes and approaching anodes with and without the use of a reducing agent, sodium bisulfite (NaHSO_3_). When NaHSO_3_ was added to the soil before electro-kinetic treatment, it decreased Cr (VI) to Cr (III) by 90.3%, as shown in [13]. The investigated efficacy of saturated soil has various alternatives for purging as follows: acetic acid (1 M AA), ethylene diamine tetra-acetic (0.1 M EDTA), sodium dodecyl sulfate (SDS), and ammonium citrate (1 MAC). These are used as a catholyte solution coupled with zeolite as a permeable reactive barrier in the electro-kinetic (EK) process (PRB), as shown in [14].

Four EK remediation experiments were conducted to compare the effects of anodes with and without a membrane. Various parameters, including current, pH, chromium removal, and changes in Cr^3+^ chemical fractionation, have been used to evaluate the process. The findings show that electro-kinetic remediation of the approaching anodes enhanced the current and reduced the sludge pH. Citric acid, as a catholyte, was more appropriate for reducing the accumulation effect and improving the Cr(total) removal rate. This study aimed to discover the effect of the membrane technique, which limits Cr accumulation after the enhancement of electro-kinetic treatment. The influence of using distilled water (DW) and a single (acetic acid (AA)) purging solution to remove Cr^3+^ from contaminated sludge was estimated, and the potential influence of the membrane in electro-kinetic remediation experiments on the removal efficiency of Cr^3+^ was assessed.

## 2. Materials

### 2.1. Contaminants

To simulate the sludge chromium contaminant, a chemical formula solution for chromium nitrate [Cr(NO_3_)_3_·9H_2_O] was used. The prepared solution was added to the specimen to obtain a representative concentration. The chemical formula for chromium nitrate [(Cr(NO_3_)_3_·9H_2_O)] used in this study had a density of 1.85 g/cm^3^, solubility in water of 81 g/100 mL (20 °C), and molecular weight of 238.011 g/mol. For example, for preparing a sludge specimen with a chromium concentration of 599.8 mg/kg and an initial moisture content equal to 40% by weight, 4.889 g of Cr(NO_3_)_3_·9H_2_O was dissolved in 400 mL of distilled water, and the solution was added to 1 kg of dried sludge, which is in accordance with the same procedure adopted by Refs. [15,16].

### 2.2. Sludge

**Figure 1:**
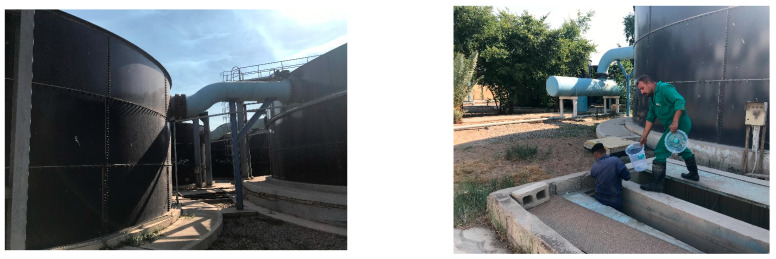
The sedimentation basins of the Kadhimiya Water Project.

The sludge utilized in this study was obtained from the Municipality of Baghdad/Baghdad Water Department/Operation Department/Kadhimiya Water Project. It was collected from clay sediments in the sedimentation basins of the Kadhimiya Water Project, as shown in Figure 1.

It was thoroughly cleaned, dried, and sifted with an extra sieve to ensure satisfactory homogeneity. Table 1 shows the physical and chemical properties of the utilized sludge.

### 2.3. Membrane

**Figure 2:**
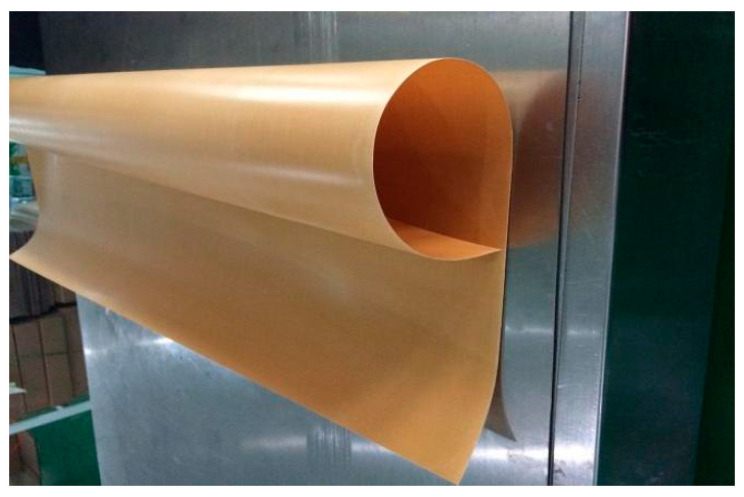
Cation exchange flat sheet membrane [17].

The anionic and cationic heterogeneous ion exchange membranes used in this study for the electro-coating process and water treatment are shown in Figure 2. Superior in terms of performance and physical stability, our cation exchange membranes are suitable for use in AED processes of all kinds. Table 2 lists the technical specifications for cation exchange flat sheet membranes.

### 2.4. Acetic Acid (AA)

The cathode chamber was flushed with acetic acid as a cleaning solution. It was composed of monoprostonic acid and acetic acid, which have the formula CH_3_COOH. In an aqueous solution, this acid partially dissociates into hydrogen ions (H^+^) and acetate ions (CH_3_COO^−^). The produced hydrogen ions (H^+^) can decrease the solution pH and dissolve metal precipitates, whereas the acetate ions may be complex with other metal ions present in the solution [18].

## 3. Experimental Setup

**Figure 3:**
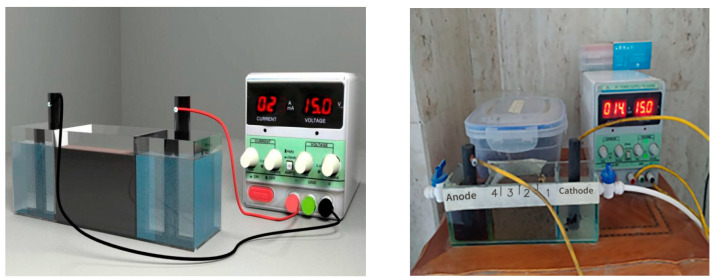
The experimental setup of the electro-kinetic cell was utilized in the present study.

Figure 3 shows the four shapes of the EK experimental setup presented in this study using electro-kinetic with and without membrane techniques. The first setup experiment consisted of a rectangular chamber made of plexiglass (7 cm D × 6 cm W × 22 cm L) and divided into three chambers, which were made of rectangular chambers in the middle (7 cm D × 6 cm W × 10 cm L) and each side (7 cm D × 6 cm W × 6 cm L), 2 electrodes, a power supply, and a multi-meter. A sludge with a height of 5 cm was created in the cells’ center. The anode and cathode were placed in the middle of the chambers on either side of the cell, creating an anode and cathode pair. The cathode and anode electrodes comprised two cylindrical graphite electrodes that were chemically inert to electrolysis operations (the diameter and length of the electrodes were 1.5 cm and 10 cm, respectively). Additional chemical species may complicate electrochemistry during processing if inert electrodes are used. Because the oxidation process has a very corrosive impact on the anode, its utilization is essential [19]. The length of the sludge specimen in this cell was determined to be five centimeters. Electro-osmotic water flow may be achieved by perforating the plexiglass surfaces of the middle chamber. The sludge could not enter the electrode compartments because the edges of the intermediate chamber were lined with filter paper.

Power cables placed into the electrodes’ tops provided continuous voltage, and a multimeter was used to monitor the voltage and quantify the flow of current through the sampled sludge, as reported in [15,20]. The second setup experiment had the same arrangement as the first one, except that the membrane had dimensions (6 cm D × 6 cm W) inserted between the filter paper and the contaminated sludge on the side surface of the middle chamber near the cathode chamber. These experiments were conducted using distilled water (Ph~10) as the catholyte.

Figure 3 also displays a schematic diagram of the setup (third and fourth) experiments using the electro-kinetic with and without membrane, with the same arrangement as the setup experiments (first and second), except that these experiments were conducted using 1M acetic acid (pH~3) as the catholyte. Because acetic acid is a weak acid that cannot strongly dissociate, it is frequently sufficient to drop the sludge pH. Consequently, adding acetic acid to the sludge would not significantly enhance its electrical conductivity [21].

**Figure 4:**
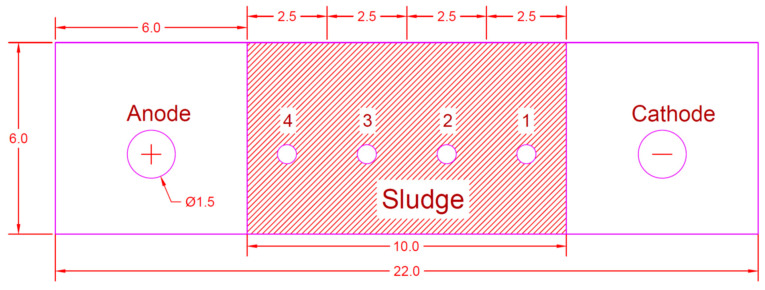
Sampling point layout.

Figure 4 shows a schematic diagram of the four shapes of the EK experiment setup: sludge sampling point (0) and the arrangement of the electrodes (C, cathode; A, anode). After the EK operation for 96 h, sludge samples were collected at the indicated points (Figure 4) using a hand auger. Then, the samples were dried, crushed, and used for analysis, as described in [22]. Table 3 summarizes the results of four experiments [Series-I, Series-II, Series-III, and Series-IV] performed using a strict methodology. The electro-kinetic process was operated with an electrode arrangement with and without a membrane (i.e., an electric field with a potential gradient of 1.5 V/cm), which were included in the experiments (EK−1, EK−2 and membrane, EK−3, and EK−4 and membrane) to study the removal of chromium from contaminated sludge.

At the end of each experiment, the sludge specimen was removed from the cell, depending on the layout of the sampling points, as described in [23] and shown in Figure 4, to determine the remaining chromium and the pH of the sludge. The distance between sampling points (1, 2, 3, and 4) was equal to 2.5 cm between each point. Sample point (4) was near the anode electrode, whereas sample point (1) was near the cathode electrode.

### Analysis of Samples

The contaminated sludge samples were analyzed to determine the amount of residual chromium ions in the sludge. One gram of dry sludge was taken and digested with an HCl/HNO_3_ solution at a ratio of 25:5 (*v*/*v*) (6). The chemical properties of the contaminated sludge sample were pH (7.8), organic matter content (OMC) (4.36%), calcium carbonate (CaCO_3_) (18.7889%), sulfate ions (SO_4_) (0.15 mg/L), and chloride content (Cl^−^) (1.099 mg/L). The electro-kinetic remediation technique obtained the average removal efficiency of total chromium according to the procedure adopted in many studies, such as Refs. [15,24,25].

## 4. Results and Discussion

### 4.1. Sludge Analyses

**Figure 5:**
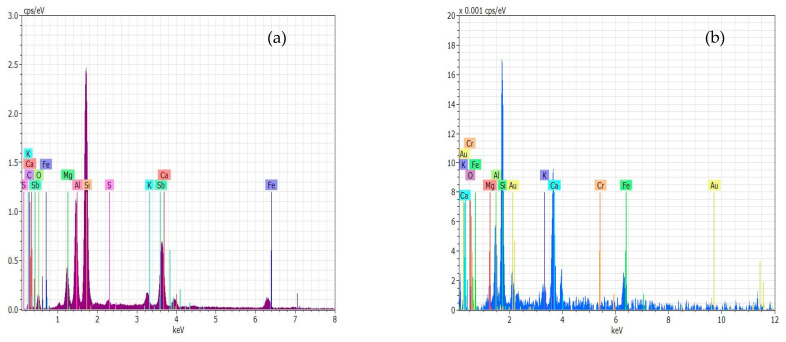
EDS for the composition, (**a**) sludge, (**b**) contaminated sludge with chromium ions.

Energy-dispersive X-ray spectroscopy (EDS) is an analytical technique that enables the chemical characterization/elemental analysis of materials. In Figure 5a,b, the EDS curves indicate that the sludge contains high levels of Si, Sb, C, Al, O, Ca, Mg, Fe, K, and S, whereas contaminated sludge contains high levels of SE, Si, Ca, Fe, K, Mg, Al, and Cr.

### 4.2. Experiment Methodology

**Figure 6:**
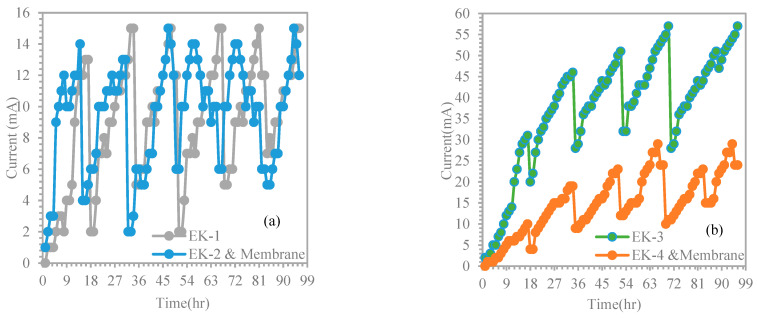
Current variation versus time: (**a**) EK−1 and EK−2 and membrane, and (**b**) EK−3 and EK−4 and membrane.

To study the effect of electric variation with time, the experimental results of EK−1 were compared with the results of EK−2 and membrane (Figure 6a). The study tests had the following conditions: initial Cr^3+^ concentration of 599.8 mg/kg, voltage gradient of 1.5 V/cm, treatment duration of 4 days, cell height of 5 cm, and using distilled water (pH~10) as the catholyte. We used a membrane when dealing with contaminated sludge in EK−2, and the use of a membrane made a noticeable difference compared to EK−1. Similar observations were made for tests EK−3 and EK−4 and membrane, except that acetic acid (pH~3) was used as the catholyte (Figure 6b). For EK−1 and EK−2 and membrane, the current gradually increased to reach high values of 15 and 12 mA compared to the current values of EK−3 and EK−4 and membrane, which were 57 and 24 mA, respectively. However, there was evidence of fluctuations in the current profile for all the tests, i.e., EK−1, EK−2 and membrane, EK−3, and EK−4 and membrane. However, an appreciable difference between the current values of EK−1, EK−2 and membrane, EK−3, and EK−4 and membrane can be recognized. This may be due to the effect of using acetic acid, which uniformly supplied the ions of the purging solution via the soil medium from the cathode to the anode electrode. Therefore, the current flowing through the soil was determined using soil conductivity, which was determined by the concentration of ionic species in the pore fluid. This suggests that a higher concentration of ions results in greater current values passing through the soil.

**Figure 7:**
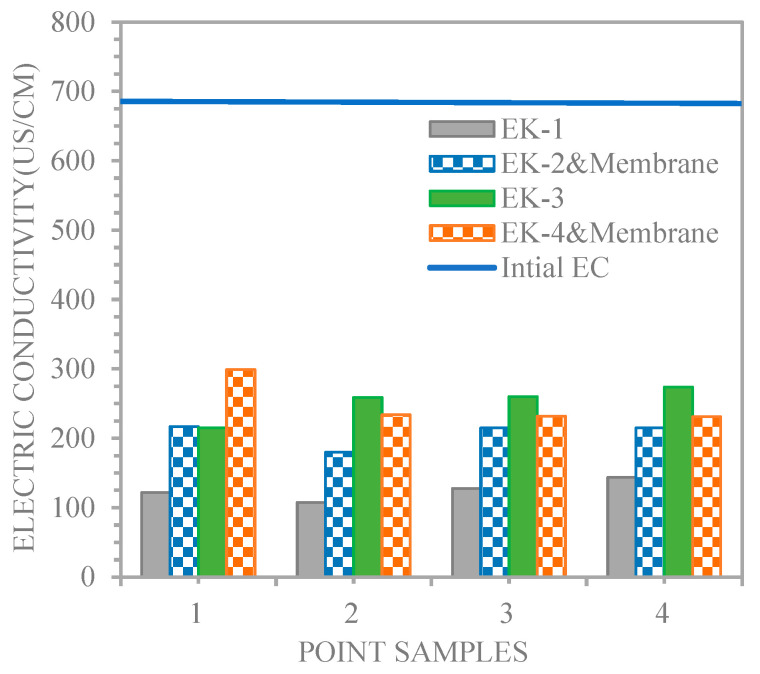
Electric conductivity profiles for the line of sampling points.

The quantity of ion electromigration is an indicator of the electric current [25]. Therefore, the electric current variations for the EK−1 and EK−2 and membrane, and EK−3 and EK−4 and membrane experiments were regarded as a function of the distances from the cathode for the lines of sample points 1, 2, 3, and 4. Figure 7 shows that the soil electrical conductivity increase in the experiments (215, 259, 260, and 274, and 299, 234, 232, and 231 μs/cm for EK−3 and EK−4 and membrane, respectively) was greater than that in the experiments (121.8, 107.5, 127.7, and 143.8, and 217, 179.8, 215, and 215 μs/cm for EK−1 and EK−2 and membrane, respectively). Saeedi et al. [21] indicated that the dissociation of some compounds, such as acetic acid, might increase the soil’s electrical conductivity during experiments. Therefore, acetic acid positively enhances the electrical current through the soil.

#### 4.2.1. Effect of Electro-Kinetic Remediation with Chromium-Contaminated Soil on the Chemical Properties

On the other hand, according to Ref. [26], the solubility of metal ions in soil depends on the metal structure and the chemical compounds, pH, and Eh (redox potential) methodologies. Scientists believe that pH is a critical factor in how soil pollutants are absorbed and mobilized. During the investigation, it was discovered that the polluted sludge had an acidic pH of 7.8, whereas the native sludge had an alkaline pH of 8.26. Sludge samples with the greatest pH buffering ability due to their high salt content, such as calcite, carbonate, or other kinds, had a modest variance in pH value. The calcium carbonate (CaCO_3_) concentrations were 25.1846% in the native sludge and 18.7889% in the polluted sludge.

**Figure 8:**
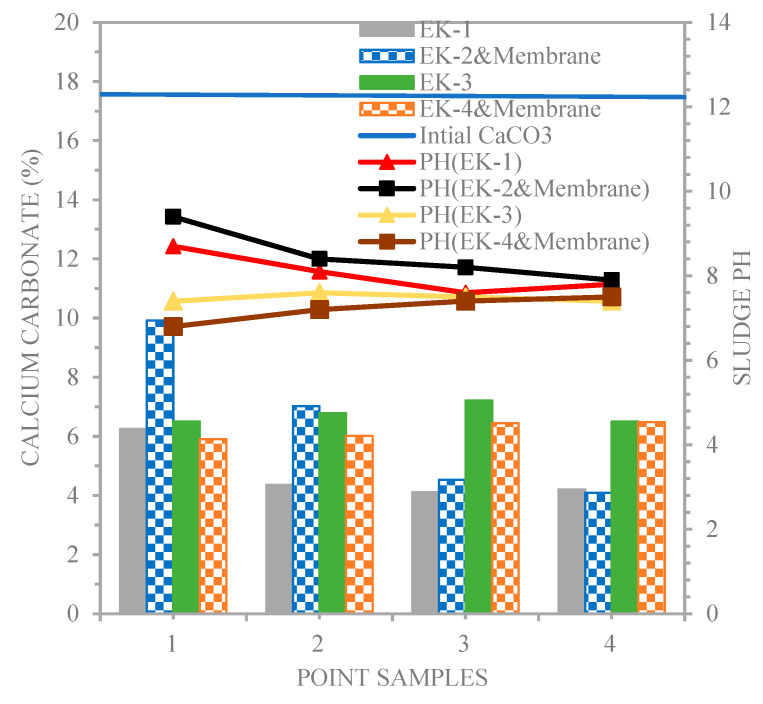
Calcium carbonate and pH profiles for the line of sampling points.

Figure 8 shows that calcium carbonate (CaCO_3_) and pH values were 6.25, 4.36, 4.11, and 4.20%, and 8.7, 8.1, 7.6, and 7.8 (EK−1), 9.9146, 7.02, 4.53, and 4.09%, and 9.4, 8.4, 8.2, and 7.9 (EK−2 and membrane); 6.5, 6.781, 7.21, and 6.498%, and 7.4, 7.6, 7.5, and 7.4 (EK−3); and 5.9, 6.01, 6.44, and 6.48%, and 6.8, 7.2, 7.4, and 7.5 (EK−4 and membrane), respectively. The anode electrode and membrane placed in the soil with a high buffering capacity (higher pH buffering capacity due to its high content of carbonate) under the action of acetic acid as a dissociated catholyte had a more remarkable input of H^+^ ions, which led to a decrease in the soil pH throughout the sampling points along the line of the sludge beginning at the cathode for EK−3 and EK−4 and membrane. This technique prevented the soil pH from reaching a high value due to the transport of H^+^ ions from one anode electrode to the cathode during the electro-kinetic technique via electro-migration. Almeira et al. [27] studied the effect of electrode configuration on the acid/basic region, minimizing the basic area and maximizing the soil’s acidity.

In Table 4, the values of chloride ions were 0.5998 mg/L (native sludge), 0.0999, 0.0999, 0.1599, 0.199 mg/L (EK−1), 0.1999, 0.4998, 0.4998, 0.5990 mg/L (EK−2 and membrane), 1.1996, 1.299, 1.299, and 1.399 mg/L (EK−3), and 1.16, 1.099, 1.099, and 1.1996 mg/L (EK−4 and membrane) sludge samples points, respectively, corresponding to a reduction of 83.3, 83.3, 73.3, 66.8% (EK−1), 66.7, 16.7, 16.7, 0.13% (Ek−2 and membrane), −100, −116.6, −116.6, −133.2% (EK−3), and −93.4, −83.2, −83.2, −100% (Ek−4 and membrane), respectively, compared with the native sludge. The reduction decreased with increasing distance from the cathode for lines 1, 2, 3, and 4 in EK−1 and EK−2 and membrane, except in EK−3 and EK−4 and membrane.

#### 4.2.2. Scanning Electron Microscopy (SEM) of Sludge Samples

**Figure 9:**
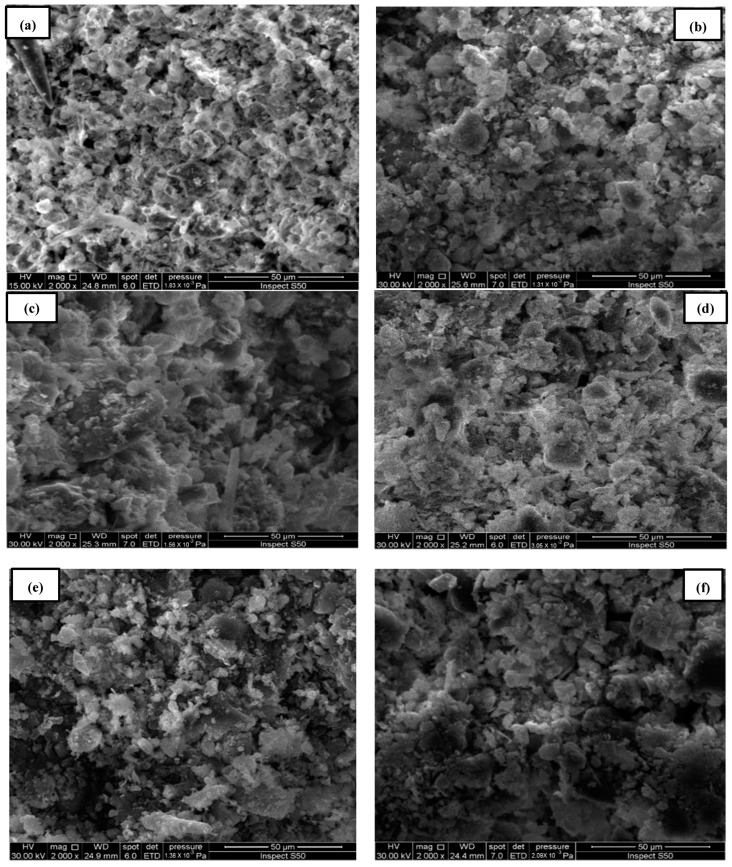
SEM images for the samples: (**a**) sludge, (**b**) contaminated sludge with chromium ions, (**c**) sludge treatment (EK−1), (**d**) sludge treatment (EK−2 and membrane), (**e**) sludge treatment (EK−3), and (**f**) sludge treatment (EK−4 and membrane).

Scanning electron microscopy (SEM) images of the sludge particles and pore structures were used to characterize the electro-kinetic treatment processes with and without membranes. The magnification of sludge, chromium-contaminated sludge, and sludge treatment (EK−1, EK−2 and membrane, EK−3, and EK−4 and membrane) was performed on a 20 μm scale using scanning electron microscopy (SEM). Figure 9 displays the morphological parameters of the sludge samples before and after the removal of chromium ions via electro-kinetic treatment operations. In Figure 9a, the sludge appears normal, whereas in Figure 9b, the sludge is contaminated with chromium ions. The sludge particles in Figure 9b were saturated with pollutant residues, resulting in the sludge surface being coated with chromium ions. [28,29].

No more pollutants were absorbed due to the reduction in sorption [30]. Compared to (EK−2 and membrane) and (EK−4 and membrane), where the membrane was added to contaminated sludge with chromium ions, the pore spacing between the sludge particles for (EK−1 and EK−3) was more significant (c, d, e, and f).

### 4.3. Distributions for pH and Chromium in the Electro-Kinetic Experiments

**Figure 10:**
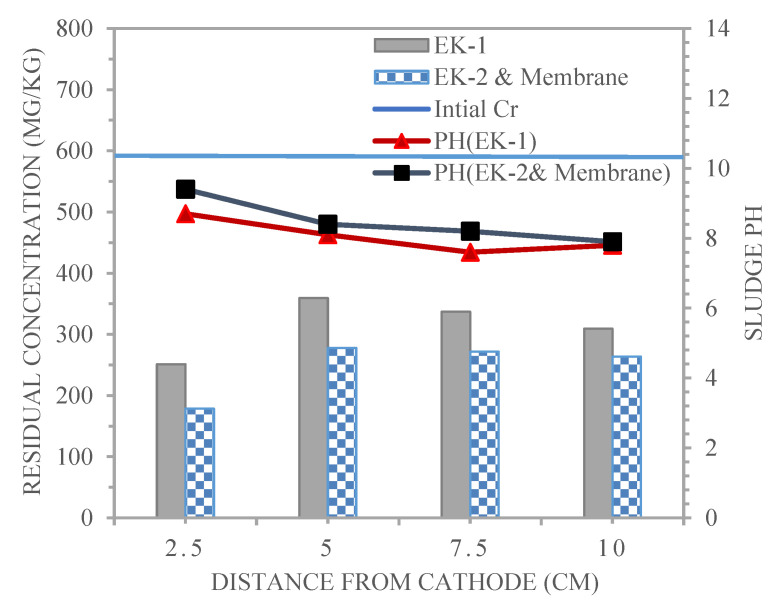
Chromium and pH profiles in the sludge treated by the electro-kinetic technique for EK−1 and EK−2 and membrane.

Figure 10 shows the distribution of Cr^3+^ content and pH detected after the end of the electro-kinetic remediation period for experiments EK−1 and EK−2 and membrane, which was 96 h, at four distances from the cathode for the lines of sample points 1, 2, 3, and 4, which were 2.5, 5, 7.5 and 10 cm. As displayed in Figure 10, the profiles of the soil pH in the EK−1 and EK−2 and membrane experiments for each line of sample points were above the background value of 7.8. In the EK−1 and EK−2 and Membrane experiments for each line of sample point 1, the sludge pH at point 1, 2.5 cm from the cathode, was 8.7 and 9.4, respectively. At middle points 2 and 3, which were located at distances of 5 and 7.5 cm from the cathode, H^+^ and OH^−^ ions were transported and encountered in the middle sections (2 and 3), with pH values of 8.1 and 7.6 and 8.4 and 8.2, respectively. The sludge pH at point 4, 10 cm from the cathode, was equal to the background pH value of 7.8. During the EK cleanup procedure, water electrolysis generated H^+^ and OH^−^ ions [31]. The sludge pH increased near the cathode region and decreased close to the anode region compared to the initial condition.

The remaining concentrations of Cr^3+^ in the sample lines obtained for the EK−1 and EK−2 and membrane experiments after remediation are shown in Figure 10, together with Table 5. The remaining concentration of Cr^3+^ in the silty clay sludge after treatment using distilled water as a purging solution is as follows:

The amount of Cr^3+^ residual for treated sludge from point 1 (near the cathode) towards point 4 (near the anode) for EK−1, equivalent to 251.3, 359.2, 337, and 309.2 mg/kg, respectively, was higher than that in EK−2 and membrane (178.4, 277.8, 271.6, and 263.4 mg/kg). It was clear that the residual concentration of Cr^3+^ at points 1, 2, 3, and 4 for EK−1 and EK−2 and membrane decreased from an initial value of 599.8 mg/kg.

It is noted that the concentration of chromium at sample points 1, 2, 3, and 4 for EK−1, as explained in Table 5, was higher than in the case for EK−2 and membrane because using membrane in the sludge achieved more efficient removal of chromium compared to EK−1, as evident from the reduction in chromium that was observed for EK−2 and membrane, which was equal to 70.2, 53.6, 54.7, and 56.1% for points 1, 2, 3, and 4, respectively, and was higher than those for EK−1 (58.1, 40.1, 43.8, and 48.4%, respectively). H^+^ from the anode and membrane was transported more rapidly to the cathode when there was a shorter distance between the two electrodes. As the rate of H^+^ migration increased, so did the rate at which chromium was desorbed and dissolved from the sludge, improving the removal impact [32].

**Figure 11:**
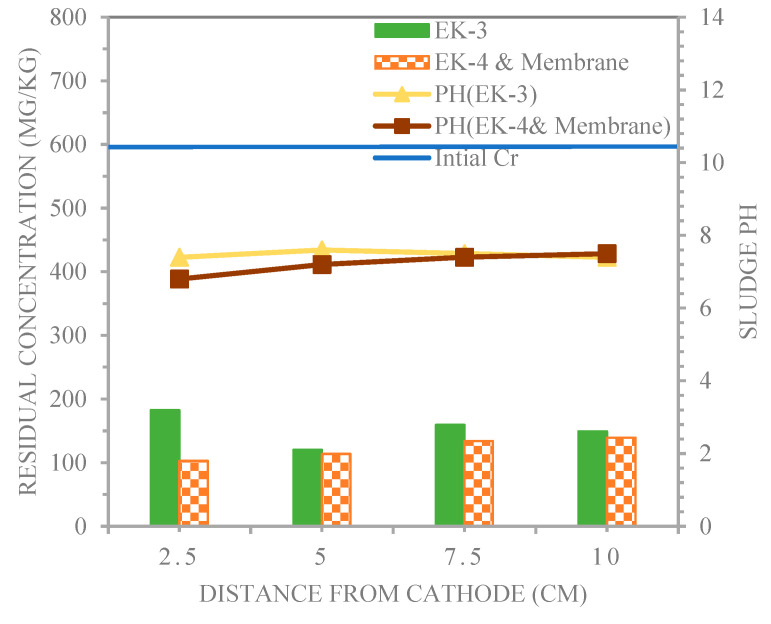
Chromium and pH profiles in the sludge treated by electro-kinetic technique for EK−3 and EK−4 and membrane.

Figure 11 illustrates the distribution of the chromium concentration and pH in the sludge after the EK−3 and EK−4 and membrane experiments in the longitudinal direction for sampling. In Figure 11, an optimal trend of an excessively low pH was formed gradually from 1, 2, 3, and 4 in EK−4 and membrane, and the pH of both sludge sample line experiments of EK−3 and EK−4 and membrane for 1, 2, 3, and 4 were 7.4, 7.6, 7.5, 7.4 and 6.8, 7.2, 7.4, and 7.5, respectively. The sludge pH ranged from 6.8 to 7.6 (below the initial pH = 7.8), and the pH of the sludge and solubility of the metal compound are of critical importance for the effective removal of Cr^3+^ from contaminated sludge [32]. These results are in agreement with those of the previous studies. Wan et al. [23] stated that the soil pH value was between 4.0 and 6.1 after 120 h. of electrodynamics restoration. This indicates that the acidity zone gradually advances throughout the soil, which is conducive to the removal of heavy metals.

Table 6 shows that the electro-kinetic processes witnessed residual concentrations of chromium of 182.6, 120.2, 159.5, and 149.1 and 102.8, 113.8, 134.1, and 139.3 mg/kg for EK−3 and EK−4 and membrane, respectively, corresponding to the effective removal efficiencies of 69.5%, 79.9%, 73.4%, and 75.1%, and 82.8%, 81%, 77.6%, and 76.8%, respectively. As a result, a low pH level was a desirable condition for metal extraction from sludge. In electro-kinetic processes, a large proportion of H^+^ ions were produced by electrolyte electrolysis from the anode. The soil around the anode was acidified so that metal ions were more easily dissolved from the sludge, dissolved in the solution, and transported by electromigration and electroosmotic flow. In addition, the ion speed of movement accelerated under acidic conditions [32].

Sludge pH at the point 1 sample locations 2.5 cm from the cathode gradually decreased (EK−4 and membrane). The sludge pH value of the electrodynamic reaction chamber ranged from 4 to 6.8 after restoration. Because the pH of the anode sludge was not controlled, an acidic migration zone progressively emerged throughout the whole batch. This allowed heavy metals to dissolve and be removed more easily. Heavy metal removal was affected by an excessively low sludge pH, which alters the polarity of the zeta potential. Therefore, the sludge pH must be maintained appropriately to ensure that heavy metal ions remain dissolved and that the soil’s negative zeta potential is maintained.

The effect of the membrane on the migration of Cr^3+^ that occurred toward the cathode was studied. For EK−4 and membrane, the residual concentration of Cr^3+^ at point 1 was relatively low, with a value of 102.8 mg/kg, compared to the residual concentration value of EK−3, which was 182.6 mg/kg. It was observed that (EK−4 and membrane) clearly outperformed (EK−3). The lower the soil pH (i.e., pH equal to 6.8 and 7.4 at point 1 for EK−4 & Membrane and EK−3, respectively), the more positive charges Cr^3+^ species have and soluble (Cr^3+^) with net cationic charges, which will speed up toward the cathode by electromigration. Therefore, high redox potential and low pH were useful to extract metal ions from all sludge fractions and expedite the electro-treatment influence, especially close to the anode, as cited in Ref. [25].

Acidity plays a critical role in the movement of metals through the soil. As the pH decreases, the metals become more mobile. They desorb from the surface of the soil $\left(M_{soil}^{2+}\right)$ to the aqueous phase $\left(M^{2+}\right)$ under equilibrium conditions [33].

(1) $$M_{soil}^{2+}+2H^{+}↔2H_{soil}^{+}+M^{2+}$$

**Figure 12:**
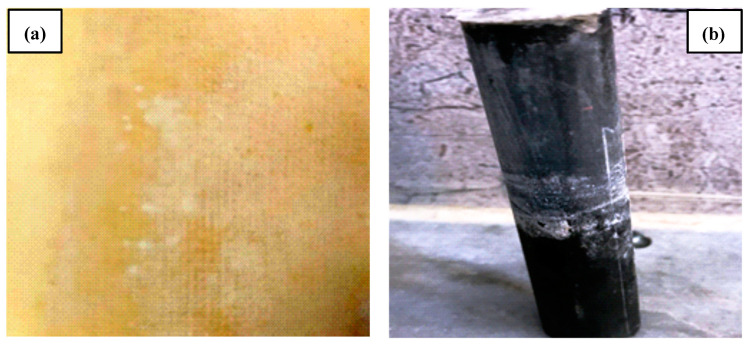
Accumulation of salts on the surface of the membrane and cathode electrodes after the end of the electro-kinetic process (EK−4 and membrane). (**a**) Membrane; (**b**) cathode electrode.

Membrane fouling is a process via which the particles, colloidal particles, or solute macromolecules are deposited or adsorbed onto the membrane pores or decreased in size onto a membrane surface via physical and chemical interactions or mechanical action, which results in smaller or blocked membrane pores. Therefore, the effects of the anode electrode and membrane were remarkable when replenishing the sludge solution system with H^+^ ions. Thus, the sludge pH for the treated sludge in the EK−4 and membrane varied from 6.8 to 7.5. Accordingly, the increasing removal of the salts from the specimen with one anode electrode and the membrane surrounding the cathode electrode can be distinguished by the large quantities of these salts on the surface of the membrane and cathode electrodes compared to other experiments, as shown in Figure 12.

## 5. Conclusions

The following are the key findings from the experimentation:

1. The electro-kinetic and membrane techniques exhibited higher Cr^3+^ removal efficiencies compared to electro-kinetic techniques performed under similar purging solution conditions.
2. The electro-kinetic process offers the advantage of using the membrane technique, in which there is no accumulation of chromium at all the sampling points of all the experiments, and this is a success in itself.
3. The membrane technique for acetic acid as a catholyte witnessed an excessively low pH of 6.8 in the EK−4 and membrane system at point 1 sampling points in the remediation of chromium-contaminated sludge. In addition to providing a higher removal efficiency using the same acetic acid, the average removal efficiencies for the EK−3 and EK−4 and membrane methods were 74.4% and 79.6% at the 1, 2, 3, and 4 sampling points, respectively.

## Figures and Tables

**Table 1:** Composition and properties of the sludge.

Property	Value
Particle size distribution	
Sand (%)	1.1
Silt (%)	63.5
Clay (%)	35.3
Cation exchange capacity (cmol·kg^−1^)	32.87
Porosity (%)	51.69
pH value	8.26
Electric conductivity, EC (μs/cm)	1500
Organic Matter (OMC %)	5.34
Calcium carbonate CaCO_3_ (%)	25.18455
Sulphate ions, SO_4_ (mg/L)	0.00514
Chloride ions, Cl^−^ (mg/L)	0.5998
Total suspended solids, TSS (mg/L)	600

**Table 2:** Flat sheet membrane samples for feasibility test [17].

Model: CE2					
Physical and Chemical Properties			U.S. Units		Metric Units
Functional group			Sulfonic acid		Sulfonic acid
Exchange capacity	min.			meq/g	1.4
Current density	max.	Ampere/ft^2^	50	Ampere/m^2^	538
Area resistance	ohm/cm	0.1 N NaCl	25	0.1 N NaCl	25
		1.0 N NaCl	10	1.0 N NaCl	10
Permeslectivity	0.5 N NaCl/1.0 N NaCl		96		96
Water permeability @5psi	max.	mL/h/ft^2^	50	mL/h/ft^2^	538
Mullen burst test	min.	psi	150	bar	10.3
Stability	pH range		1–10		1–10
Stability	max. temp	℉	176	°C	80
Dimensions	max. width	inches	43	meters	1.09
Dimensions	max. length	inches	122	meters	3.1
Dimensions	approx. thickness	mils	20	mm	0.51
Ionic form as shipped			Na^+^		Na^+^
Storability	of products	max. years	2	max. years	2
Storability	temp range	℉	40–75	°C	4–24

**Table 3:** Electro-kinetic remediation experiments are described in detail.

Series	Experiment Designation	Conc. (mg/kg)	Time (h)	PS (pH)		Electrodes Arrangement	Membrane
				Cathode	Anode		
Series I	EK−1	599.8	96	DW	DW	One Cathode + One Anode	----
Series II	EK−2 and Membrane	599.8	96	DW	DW	One Cathode + One Anode	Membrane
Series III	EK−3	599.8	96	AA	DW	One Cathode + One Anode	----
Series IV	EK−4 and Membrane	599.8	96	AA	DW	One Cathode + One Anode	Membrane

DW: Distilled water. AA: Acetic acid.

**Table 4:** The concentration of chloride ions for various sludge samples.

Sludge Samples	Chloride Ions (mg/L)							
	EK−1		Ek−2 and Membrane		EK−3		Ek−4 and Membrane	
Native Sludge	0.5998	Reduction (%)	0.5998	Reduction (%)	0.5998	Reduction (%)	0.5998	Reduction (%)
1	0.0999	83.3	0.1999	66.7	1.1996	−100	1.16	−93.4
2	0.0999	83.3	0.4998	16.7	1.299	−116.6	1.099	−83.2
3	0.1599	73.3	0.4998	16.7	1.299	−116.6	1.099	−83.2
4	0.199	66.8	0.5990	0.13	1.399	−133.2	1.1996	−100

**Table 5:** Residual Con. of chromium for sampling points at the end of the electro-kinetic treatment using distilled water.

Parameters	Points of Samples (EK−1)				Points of Samples (EK−2) **& Membrane**			
	1	2	3	4	1	2	3	4
Initial Con. of Cr(II)	599.8 mg/kg							
Con. of Cr(II) mg/kg	251.3	359.2	337	309.2	178.4	277.8	271.6	263.4
Reduction (%)	58.1	40.1	43.8	48.4	70.2	53.6	54.7	56.1
Average (%)	47.6				58.6			

**Table 6:** Residual Con. chromium for sampling points at the end of the electro-kinetic treatment using distilled water and acetic acid as the purging solution.

Parameters	Points of Samples (EK−3)				Points of Samples (EK−4) **and Membrane**			
	1	2	3	4	1	2	3	4
Initial Con. of Cr(II)	599.8 mg/kg							
Con. of Cr(II) mg/kg	182.6	120.2	159.5	149.1	102.8	113.8	134.1	139.3
Reduction (%)	69.5	79.9	73.4	75.1	82.8	81	77.6	76.8
Average (%)	74.4				79.6			

## Data Availability

The authors declare that all data supporting the findings of this study are available within the article.
